# Collagens Regulating Adipose Tissue Formation and Functions

**DOI:** 10.3390/biomedicines11051412

**Published:** 2023-05-10

**Authors:** Iida Jääskeläinen, Tiina Petäistö, Elahe Mirzarazi Dahagi, Mahdokht Mahmoodi, Taina Pihlajaniemi, Mari T. Kaartinen, Ritva Heljasvaara

**Affiliations:** 1 ECM-Hypoxia Research Unit, Faculty of Biochemistry and Molecular Medicine, University of Oulu, 90014 Oulu, Finland; 2 Department of Anatomy and Cell Biology, Faculty of Medicine and Health Sciences, McGill University, Montréal, QC H3A 0C7, Canada; 3 Faculty of Dental Medicine and Oral Health Sciences, McGill University, Montréal, QC H3A 0C7, Canada; 4 Division of Experimental Medicine, Faculty of Medicine and Health Sciences, McGill University, Montréal, QC H3A 0C7, Canada

**Keywords:** adipogenesis, basement membrane, collagen, diabetes, dyslipidemia, extracellular matrix, fibronectin, fibrosis, lipodystrophy, obesity

## Abstract

The globally increasing prevalence of obesity is associated with the development of metabolic diseases such as type 2 diabetes, dyslipidemia, and fatty liver. Excess adipose tissue (AT) often leads to its malfunction and to a systemic metabolic dysfunction because, in addition to storing lipids, AT is an active endocrine system. Adipocytes are embedded in a unique extracellular matrix (ECM), which provides structural support to the cells as well as participating in the regulation of their functions, such as proliferation and differentiation. Adipocytes have a thin pericellular layer of a specialized ECM, referred to as the basement membrane (BM), which is an important functional unit that lies between cells and tissue stroma. Collagens form a major group of proteins in the ECM, and some of them, especially the BM-associated collagens, support AT functions and participate in the regulation of adipocyte differentiation. In pathological conditions such as obesity, AT often proceeds to fibrosis, characterized by the accumulation of large collagen bundles, which disturbs the natural functions of the AT. In this review, we summarize the current knowledge on the vertebrate collagens that are important for AT development and function and include basic information on some other important ECM components, principally fibronectin, of the AT. We also briefly discuss the function of AT collagens in certain metabolic diseases in which they have been shown to play central roles.

## 1. Adipose Tissues

Adipose tissue (AT) is a type of loose connective tissue that functions as an energy reservoir and protects other tissues and organs from lipotoxicity. AT is also an important endocrine system that secretes adipokines (adipo-cytokines), making it a key tissue that regulates whole-body lipid and glucose metabolism as well as general health [1,2]. There are at least three types of ATs: white AT (WAT), brown AT (BAT), and beige or brite (brown-in-white) AT [1]. Furthermore, bone marrow adipose tissue (BMAT) has been suggested to form its own distinct type of AT, while lactating breast tissue is described to include pink adipocytes [2]. In humans, BAT depots localize mainly in the cervical-supraclavicular and perirenal regions of the body and are most prominent in infants and young adults, and decrease upon aging. The largest AT, the WAT, can be divided into two main depots based on its anatomical location, function, and signaling. Subcutaneous AT (SAT) is located directly under the skin and visceral AT (VAT) localizes around internal organs in the abdominal cavity [2,3].

Morphologically, white adipocytes are characterized by one large lipid droplet which presses the nucleus and cell organelles against the cell membrane, which reflects their obvious function of fat storage [4]. The properties of white adipocytes can differ depending on their location. VAT adipocytes are larger and metabolically more active, more prone to lipogenesis and lipolysis, and release more pro-inflammatory cytokines. In contrast, SAT produces higher levels of favorable adipokines, which include adiponectin and leptin, and is more vascularized and less vulnerable to the adverse effects of obesity. While both types of depots expand in obesity, an increase in VAT around internal organs is more detrimental to metabolic health. Increased VAT also correlates with metabolic dysfunctions [5].

Brown adipocytes are functionally and morphologically distinct from white adipocytes. They are smaller in size than white adipocytes and have a multilocular lipid reservoir [4]. While WAT stores lipids, BAT consumes fatty acids for heat production (thermogenesis) by disengaging the respiratory chain from energy production in the mitochondria via a brown-adipocyte-specific protein called uncoupling protein 1 (UCP1) [2,4,6]. Beige adipocytes are spread amongst WATs, and, upon activating signals, such as a cold temperature, they upregulate the production of brown-adipocyte-specific proteins and begin to resemble brown-like cells [7]. After the identification of physiologically active BAT in adult humans [8,9,10], its activation as a therapeutic strategy for the treatment of obesity and related disorders has attracted substantial interest. In cell and animal models, the transdifferentiation or browning of white adipocytes, for example via cold-, nutrient-, or noradrenaline-stimulated upregulation of UPC1, promotes energy expenditure, reduces adiposity, and protects against diet-induced obesity and insulin resistance. In humans, the pharmacological activation of BAT combined with physical exercise and a healthy diet is the most promising strategy to control weight gain [1,4,11,12,13,14].

### 1.1. Pathological Conditions in AT

**Figure 1:**
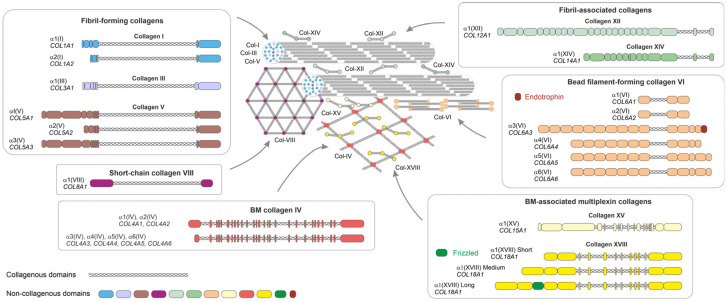
Structure and supramolecular assembly of the collagen types reported to contribute to AT physiology and pathology. Domain organizations of collagen α chains of different subfamilies are depicted in boxes. Collagens I, III, and V form collagen fibrils. Collagens XII and XIV bind to the fibrils and regulate their organization. Collagen XVIII binds to collagen IV network in the BM. Collagen XV resides at the interphase of the BM and collagen fibrils. Collagen IV forms beaded filaments at the fibrillar matrix-BM interphase. Collagen VIII forms hexagonal lattices at the BM. Triple helix, collagenous domains. Non-collagenous domains of different collagens are shown with different colours. Endotrophin domain of collagen VI and frizzled domain of collagen XVIII are indicated. The sizes of collagens and their assemblies are not presented at their correct scale. Post-translational modifications such as glycosaminoglycan chains in collagens XII, XIV, XV, and XVIII are not illustrated.

In this review, we focus on the roles of collagens (Figure 1) in the pathological conditions that primarily affect WAT. We performed a search of the literature on collagen family members in AT and adipogenesis, and in metabolic disorders including obesity, metabolic syndrome, T2D, AT fibrosis, and dyslipidemias. We summarize the current findings in the following chapters. In addition to these disorders, many other metabolic diseases are associated with obesity and dysfunctions of AT. For example, ectopic lipid accumulation in the liver, which causes non-alcoholic fatty liver disease, as well as cardiovascular diseases, renal dysfunction, infertility, and many types of cancer, are often consequences or comorbidities of problems in AT functions [15]. The roles of ECM and collagens in these diseases are discussed in several other review articles [16,17,18,19,20,21], and, therefore, are not addressed in this article.

#### 1.1.1. Obesity and Type 2 Diabetes

Obesity is characterized by abnormal and excessive fat accumulation and is a risk factor for many pathological conditions and diseases, such as insulin resistance, type 2 diabetes (T2D), and cardiovascular diseases [15]. AT accommodates to the increased requirement for fat storage in obesity via adipocyte differentiation (adipogenesis and hyperplasia) and enlargement (hypertrophy) [22]. The expanding WAT accumulates many types of immune cells, which, together with hypertrophic adipocytes, secrete pro-inflammatory cytokines that sustain persistent low-grade inflammation. This leads to harmful changes in the metabolism and gene expression profiles of adipocytes [15]. Chronic inflammation drives the development of T2D, where adipocytes do not respond to insulin signaling and, as a result, the uptake of glucose from the circulation via insulin-sensitive glucose transporter 4 (GLUT4) is reduced [23]. Insulin inhibits lipolysis in healthy WAT; however, in T2D, this pathway is dysregulated. Reduced insulin sensitivity leads to the increased breakdown of stored lipids, increases the lipid supply to circulation, and enhances the accumulation of fat to internal organs such as liver, which further complicates metabolic problems [24].

#### 1.1.2. AT Fibrosis

AT expansion involves an acute remodeling of the extracellular matrix (ECM) to allow for larger adipocytes and vascular growth in the tissue (angiogenesis) [25,26]. In obesity, the rapid growth of AT mass is associated with limited oxygen supply due to defects in tissue vascularization. The resulting hypoxic tissue environment activates the hypoxia-inducible factor-1 (HIF-1) pathway, which induces transcriptional programs that sustain AT inflammation and fibrosis. Infiltrated macrophages, as well as adipocytes, secrete pro-inflammatory chemokines and growth factors, such as tumor necrosis factor alfa (TNF-α), interleukin 6 (IL-6), and transforming growth factor beta (TGF-β), which further drive the immune progenitors toward pro-inflammatory phenotypes. Hypoxia and inflammation upregulate the expression of ECM genes, including collagens, fibronectin (FN), and hyaluronan, as well as ECM crosslinking enzymes lysyl oxidase (LOX) and transglutaminases, which leads to the excessive accumulation and rigidity of ECM elements in obese AT [25,26,27,28,29,30,31,32,33]. The stiff, fibrotic tissue environment in AT is believed to limit adipocyte size growth [34]. Therefore, the excess lipids may accumulate as ectopic fat depots elsewhere in the body, including in the liver, skeletal muscle, and other organs, as well as in the bloodstream [15]. AT fibrosis also promotes insulin resistance, and AT ECM has been suggested to play a major role in defining how severely obesity impacts the metabolic health of an individual [35,36].

Fibrosis manifests as large collagen I and III bundles which are deposited in AT, replacing the functional AT parenchyma, as well as pericellular fibrosis gathering around the adipocytes [37]. Furthermore, fibrotic bundles can also concentrate around blood vessels, leading to compromised angiogenesis [37,38]. The suppression of angiogenesis is associated with impaired tissue oxygenation and, thus, with an increase in the negative effects of AT fibrosis on metabolic health [30,39,40]. In mice, a high-fat diet (HFD) induces a transcription signature in VAT adipocytes, characterized by the upregulation of genes for ECM and cytoskeletal proteins which, together, cause the cells to experience mechanical stress from both inside and outside [41]. This leads to the promotion of fibrotic processes, and at the same time, the suppression of adipocyte programs, including many lipolytic genes and mitochondrial genes [41]. In fact, AT fibrosis involves a switch in the adipocyte phenotype towards a more fibrotic gene expression profile [41,42]. For example, macrophage-derived platelet-derived growth factor receptor α (PDGFRα) is a known pro-fibrotic signal which directly activates adipocytes and control ECM dynamics [40,43,44]. Recently, the Hippo pathway has been indicated as a key regulator of AT fibrosis in mice. In cooperation with TGF-β activity, inactivation of the Hippo pathway appears to induce AT fibrosis by promoting a shift in the adipocyte gene expression profile toward a myofibroblast-like gene expression [45].

#### 1.1.3. Lipodystropy

Lipodystrophies, also known as lipoatrophies, refer to diseases characterized by a loss of AT [46]. Lipoatrophy can be generalized, taking place in all parts of the body, or can be a local phenomenon that occurs in a certain fat depot or depots. The disease can be either genetic or, sometimes, acquired as a consequence of, e.g., autoimmune disease or HIV infection. The severity of lipodystrophies can vary depending on the type of condition and how much AT has been lost. Often, they are accompanied by severe metabolic complications such as dyslipidemias, in which the circulating lipid balance is disturbed, and insulin resistance and diabetes ensue [46]. The fact that obesity and lipodystrophy can cause similar metabolic complications highlights the importance of maintaining a balance where an appropriate amount of properly functioning AT maintains an individual’s metabolic health.

## 2. Extracellular Matrix in AT

Comparisons between lean, healthy AT and obese, unhealthy AT have revealed that healthy AT is characterized by a high gene expression and abundance of ECM elements such as collagens and ECM remodeling enzymes. In contrast, metabolically unhealthy AT presents a significant upregulation of factors that increase the rigidity of the tissue, including the collagen-crosslinking LOX, the transglutaminases TG2 and Factor XIII-A [47,48,49,50,51,52], and the cell adhesion-related TSP-1 [53]. Interestingly, in an in vitro setting, the decellularized ECM of non-diabetic AT was shown to retain the functions of the adipocytes of T2D patients, and vice versa: ECM extracted from diabetic AT had adverse effects on the adipocytes of non-diabetic individuals, indicating that ECM plays a key role in adipocyte function in diabetes [54]. Moreover, different AT depots, specifically SAT and VAT depots, have their own composition of ECM molecules which are differently up- or downregulated in obesity [53,54,55]. For example, in mice fed with a HFD, the expression of genes for collagen I and IV α chains are upregulated specifically in SAT, whereas collagen III and FN are upregulated in both SAT and VAT [54]. The fact that these depots have very different functional roles in whole-body metabolism suggests that the influence of ECM on AT functions is fundamental.

The cellular components of AT consist of multiple cell types, including preadipocytes, mesenchymal stromal cells, macrophages, immune cells, and fibroblasts. AT cells, as well as tissue structure and function, require an appropriate composition of the ECM, which is achieved through dynamic ECM remodeling, involving both its removal and the de novo synthesis of new ECM components. ECM modification is accomplished by regulating the synthesis and assembly of the ECM components themselves or by influencing the factors associated with their degradation, such as matrix metalloproteinases (MMPs) and their inhibitors. The ECM in ATs consists mainly of collagens, especially collagen types I, IV, and VI, as well as FN and laminins [34,56]. Many ECM molecules are produced by adipocytes themselves, but a major portion of the collagens are synthesized by the cells in the stromal vascular fraction [40,41].

For adipocytes, the remodeling of the ECM constitutes an important process for preadipocyte maturation. When adipocytes achieve maturity, they surround themselves with a basal lamina, or basement membrane (BM), which is a sheet-like specialized form of the ECM. The main components of the BM are laminins and collagen IV, which both form their own network and are attached to each other by various linking molecules such as nidogen [57,58]. The basal lamina helps to keep the cells intact when they are subjected to the mechanical stretch caused by their large lipid-storage droplets. In addition to providing this structural support, ECM molecules are also essential regulators of many AT functions such as energy metabolism via storing and releasing various growth factors [58,59,60]. The roles of major AT collagens in adipogenesis and AT-related disorders are further discussed in subsequent chapters.

Other important ECM proteins in the AT include FN, which is found abundantly together with fibrillar collagen ECM in the interstitial matrix as well as in the ECM of the BMs [61,62], and whose presence has been described in human and mouse WAT. The FN matrix can regulate numerous cellular functions in tissues, including cell adhesion, migration, proliferation, and differentiation [61,63,64], as well as the activity and assembly of other ECM components such as LOX [65], bone morphogenetic protein 1 (BMP-1) [66,67], collagens I and III [68,69], thrombospondin-1 (TSP-1) [68], and fibrillin-1 [70,71], at least in vitro. FN produced by tissue-resident cells is referred to as cellular FN (cFN) and contains extra domains A (EDA) and B (EDB) as a result of the alternative splicing of the *FN1/Fn1* gene [72,73]. The soluble plasma FN (pFN) is a product of the hepatocytes in the liver, from where it is secreted into the blood and circulates at a high concentration. pFN can affect several tissues and organ ECMs [74].

The FN matrix inhibits preadipocyte differentiation to mature adipocytes by maintaining cellular adhesions and fibroblastic preadipocyte morphology [75,76,77]. In preadipocyte cultures, FN is expressed at the early pre-adipocyte stage, and its role is associated with the maintenance of the pre-adipocyte phenotype via the preadipocyte factor 1 (Pref-1) [78]; however, it is not established if the form involved in pre-adipocyte maintenance is pFN or cFN. pFN is a well-known substrate for Factor XIII-A transglutaminase, which promotes its self-assembly and crosslinking to collagen type I [79,80]. This covalent modification stabilizes the pFN matrix (but not the cFN matrix) in 3T3-E1 preadipocytes, which, in turn, modulates insulin signaling [52]. Knockout mice of FXIII-A, which are protected from HFD-induced insulin resistance and inflammation, have less pFN and a less collagenous matrix in their WAT compared to their wild type controls [51]. Obese HFD-fed mice show a significant increase in the circulating EDA-FN (cFN), which, possibly through interaction with toll-like receptor 4, may mediate the development of insulin resistance in mice [81].

The SAT and VAT of obese humans were shown to have decreased *FN1* mRNA expression compared to lean control tissues and presented a negative correlation with the body mass index (BMI). However, in a lean–obese monozygotic twin study, it was also shown that *FN1* mRNA was increased in isolated SAT adipocytes in the heavier, obese twin and correlates with pre-diabetic markers [47]. The circulating pFN was found to be higher in obese individuals with normal or fatty livers [82,83].

Another notable matrix molecule in the AT is the ECM glycoprotein osteopontin (OPN). In AT, it is produced by mature adipocytes as well as by stromal cells: both macrophages and senescent T cells [84,85,86]. *OPN* expression is significantly increased in obese and overweight patients, and even moreso in patients with obese-induced T2D, as compared with lean subjects [85]. In mice fed with a HFD, OPN deficiency leads to numerous beneficial outcomes including lower body weight, better insulin sensitivity, decreased hepatosteatosis, decreased AT fibrosis, and improved BAT function [87,88]. In addition, in the absence of OPN, both systemic and AT inflammation are decreased. The mechanisms of OPN action in AT are related to ECM remodeling, as demonstrated by reduced MMP and TGF-β production in AT, as well as to the regulation of inflammation, shown by a reduced infiltration of macrophages and monocytes into AT deficient of OPN [87,88]. These findings suggest that OPN has a role in linking obesity and the development of insulin resistance.

OPN mediates signals via integrins, and via the CD44 receptor, which can also bind another common ECM molecule, the glycosaminoglycan hyaluronan (HA) [89]. HA affects, for example, monocyte recruitment into AT, as well as the process of adipogenesis [90,91]. It has been reported to inhibit the differentiation of preadipocytes in vitro, either by knocking down or overexpressing HA synthases in the culture system [92,93,94]. In in vivo mouse models, HA accumulated in AT with HFD, and exogenous HA-degrading hyaluronidase or HA synthesis inhibitor reduced VAT accumulation and hepatosteatosis and increased insulin sensitivity [91,94]. Worth mentioning as an example of an ECM molecule of VAT is also TSP-1, a glycoprotein that contributes to obesity and insulin resistance [95,96]. In addition to these, MMPs and their inhibitors, tissue inhibitors of metalloproteinases (TIMPs) that control the degradation and turnover of collagens and other ECM components, play important and variable roles in AT formation and function [59].

## 3. AT Collagens and Their Roles in Adipogenic Differentiation and Dysfunctional AT

### 3.1. Collagens in Adipogenesis

**Figure 2:**
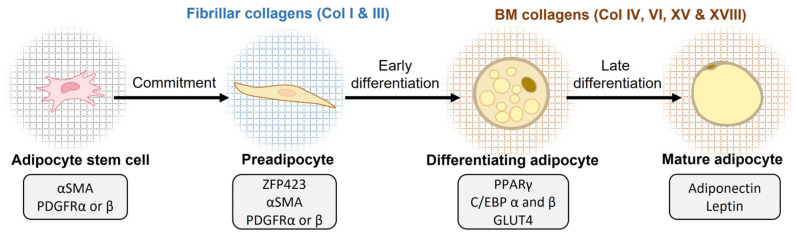
Key collagens associated with white adipocyte differentiation. During the adipogenic differentiation of adipocyte stem cells to mature adipocytes, the expression of collagens shifts from the fibrillar collagens I and III to the BM-associated collagens IV, VI, XV, and XVIII. This view is largely based on the in vitro model of murine 3T3-L1 preadipocytes, as discussed in the main text. The gray boxes present some common cell markers expressed at different stages of adipocyte differentiation. Abbreviations: αSMA—α smooth muscle actin; BM—basement membrane; C/EBP—CCAAT/enhancer-binding proteins; Col—collagen; GLUT4—glucose transporter 4; PDGFR—platelet-derived growth factor receptor; PPARγ—peroxisome proliferation-activated receptor γ; ZFP423—Zinc-finger protein 423.

Adipogenesis is the process of adipocyte differentiation from fibroblast-like precursor cells to lipid-filled mature adipocytes [22,97,98,99,100]. The first stage in the two-stage process of adipogenesis is the commitment of mesenchymal stem cell-derived adipose progenitor cells to the adipocyte lineage (Figure 2). The commitment step concludes with the formation of a preadipocyte, which still has the outer appearance of a fibroblast and expresses common fibroblast and adipose progenitor cell markers, such as α smooth muscle actin (αSMA) and PDGFRα and PDGFRβ [22]. The expression of Zinc-finger protein 423 (ZFP423) by the preadipocytes promotes adipogenic differentiation by sensitizing the cells to the BMP signal, which facilitates adipogenic progression [101]. Peroxisome proliferator-activated receptor gamma (PPARγ), together with the CCAAT/enhancer-binding protein alpha (C/EBPα), are widely accepted as the master regulators of adipocyte differentiation [22,102,103]. In the second stage, differentiating adipocytes lose their cuboidal shape and change to a morphologically round mature adipocyte shape and accumulate lipid droplets [22]. Adiponectin and leptin hormone secretion indicates that the adipocyte has fully matured. ECM attachments play a major role in regulating the pre- and mature adipocyte shape and size [22,99,102].

Decades ago, an electron microscopic study suggested the need for a three-dimensional collagen fibril-rich environment for proper adipocyte differentiation and maturation [104]. Later, a widely used in vitro model of adipogenesis, the 3T3-L1 murine preadipocytes, confirmed that, during the differentiation process from fibroblasts to adipocytes, the gene expression profiles of collagens shift from fibrillar to BM type collagens (Figure 2). During the undifferentiated fibroblast stage, the 3T3-L1 cells generally express the fibrillar collagen types I, III, and V, whereas collagen types IV and VI are expressed when they differentiate into mature adipocytes [105]. Interestingly, this shift is accompanied by changes in the expression and modification of cytoskeleton components and integrin-attachment modes. Further, the cell morphology changes from the elongated shape of pre-adipocytes to the round one of adipocytes [105] (Figure 1); this is believed to be driven by the change from fibrillar collagen and FN adhesion and signaling through integrin α5β1 to collagen IV and laminin adhesion/signaling through integrin α6β1. In addition to the morphological transformation, the transcription profiles of the cells are also impacted by the different cues from the different ECMs associated with preadipocytes and mature adipocytes [106,107,108]. In summary, the collagenous matrix surrounding the adipocytes is actively modified during the differentiation process (Figure 1) and impacts the regulation of adipogenesis during development as well as in AT expansion [22,56,109].

### 3.2. Collagens in Dysfunctional AT and Metabolic Diseases

Many of the 28 different vertebrate collagen types [60,110] are linked with various metabolic diseases in which AT is affected. In Table 1, we have summarized the current data on the collagens that are expressed in pathological AT and that are suggested to contribute to AT dysfunction and/or metabolic diseases. These collagens and their roles in adipogenesis and pathological AT are discussed in the subsequent paragraphs. To date, and to the best of our knowledge, no detailed data have been reported on the function of collagen types VII, X, XI, XVI, XVII, XIX–XXIII, and XXV–XXVIII in relation to AT and the pathological conditions associated with it; although, transcriptome analyses have revealed changes in the expression of some of these collagens upon adipogenic differentiation in vitro or in obese versus lean individuals, as discussed in Section 3.9.

### 3.3. Collagen I

Collagen I belongs to the subfamily of fibrillar collagens and is the most common collagen type in vertebrates and an important structural component in multiple tissues. In healthy tissues, collagen I molecules usually occur as heterotrimers which are composed of two α1 polypeptide chains and one α2 chain, encoded by *COL1A1* and *COL1A2* genes in humans, respectively [110] (Figure 1). Collagen I synthesis is high in AT in general, but it is enriched in SAT compared to VAT in healthy rodents. In contrast, in healthy humans, the expression levels of *Col1a1* do not vary significantly between the two depots [53,132]. Its expression is high during the early stages of adipogenesis both in vivo and in the 3T3-L1 in vitro model; however, in rat VAT, the expression decreases when the adipocytes mature and the collagen gene expression profile shifts from fibrillar collagens toward BM-associated collagens (Figure 2). In SAT, collagen I synthesis does not diminish during adipocyte maturation [105,132,133].

Remodeling of the collagen I scaffold is essential for the proper differentiation and functioning of adipocytes. For example, the degradation of collagen I by the membrane-tethered matrix metalloproteinase 1 (MT1-MMP), also known as MMP14, is crucial for the differentiation of WAT (but not BAT) [134]. Without the action of this protease, the preadipocytes are entrapped within a dense fibrillar collagen meshwork that compromises the proper tissue architecture and signaling required for adipocyte differentiation. In the *db*/*db* animal model of T2D, the collagen I synthesis rate is notably increased in WAT [34], while in fibrotic SAT and VAT of obese humans, collagen I is typically found as fibrous bundles of various thicknesses alongside collagen III [37]. Macrophage-driven stimuli were shown to induce collagen I as well as other ECM proteins [29]. Further, constitutively active HIF-1α in a transgenic mouse model resulted in an increased expression of the genes of fibrillar collagens and their crosslinking enzymes, as well as increased local inflammation, leading to AT fibrosis and dysfunction [30]. Last, HFDs were shown to further stimulate collagen I production in *db/db* mice [135]. Differing from T2D and obesity, the serum biomarkers of collagen I were downregulated in patients with T1D with retinopathy. A decreased amount of collagen I was suggested to reduce the vascular integrity in such patients [111].

Although collagen I is often considered to be a primarily structural collagen, certain intriguing functional properties have been observed for it in relation to AT as well. The coating of collagen I has been reported to promote the migration and proliferation of undifferentiated mouse 3T3-L1 preadipocytes in vitro. Collagen I may induce these effects via reactive-oxygen-species generation and the activation of p65-dependent NF-κB signaling [136]. Another explanation for collagen I-induced adipocyte migration is that it occurs due to the activation of the Hippo/YAP pathway, which promotes primary cilia growth, leading to increased 3T3-L1 cell migration [137]. However, the effect that collagen I has on preadipocyte differentiation seems to be detrimental instead. Further, 3T3-L1 preadipocytes cultured on collagen I present an increased *YAP* expression, which leads to a reduction in the synthesis of adipogenic factors, such as PPARγ and C/EBPα, and the inhibition of adipocyte maturation [138]. Another study showed that interaction between collagen I and aortic carboxypeptidase-like protein, which is a secreted protein that is highly expressed in preadipocytes but downregulated during adipogenesis, leads to a reduced expression of these adipogenic factors, thus providing evidence of ECM-derived cues that influence adipogenic differentiation [139]. Moreover, the collagen I coating was found to repress autophagy in adipocytes through YAP activation and increase their mitochondrial content, causing adipogenesis inhibition and accelerated energy metabolism in 3T3-L1 cells, which was verified by an enhanced glucose uptake, reduced fatty acid release, and increased ATP production [140,141].

### 3.4. Collagen III

Collagen III is another abundant fibrillar collagen that is found in normal tissues in the same stromal areas as collagen I [110], as well as in the fibrotic ATs of obese humans [37] (Figure 1). However, in all types of mouse fat depots, collagen III synthesis appears to be significantly lower compared to collagen I synthesis [34]. Similar to collagen I, collagen III is also upregulated in mice fed with a HFD, or in mice that experience the constitutive expression of *Hif1a* [30,135]. In patients with T1D-induced retinopathy, unlike collagen I, elevated collagen III levels were reported [111]. Further, patients with T2D-induced nephropathy present increased levels of type III procollagen aminopeptide in their blood, thus suggesting that this peptide could be a possible biomarker for diabetic nephropathy [113].

Collagen III is downregulated during the differentiation of 3T3-L1, but the depletion of collagen III in 3T3-L1 preadipocytes was shown to prevent adipogenesis, indicating that it serves an important function in this process [133,142]. Collagen III was suggested to convey its effects through its established binding partner, the G protein-coupled receptor 56 (GPR56), as the phenotype of 3T3-L1 cell with CRISPR/Cas9-mediated *Col3a1* knockout strongly resembled the phenotype of GPR56 knockout cells. The *Col3a1* knockout led to a decrease in the adipogenic markers PPARγ, C/EPBα, and aP2, and reduced cell adhesion and lipid accumulation while maintaining constant canonical Wnt/beta-catenin activity [142], which is known to impair adipogenesis [143]. The knockout of *Col3a1* also downregulated the expression of several ECM transcripts during the in vitro differentiation process, including collagens IV and VI [142]. Interestingly, the *Col3a1* deficiency also downregulated the expression of *FN* in undifferentiated 3T3-L1 cells but upregulated its expression in mature adipocytes, suggesting that the functions of these ECM components are interlinked [142].

### 3.5. Collagen IV

The non-fibrillar collagen IV is a heterotrimer that is composed of various combinations of six different α chains, and these subtypes are often classified into their own category of BM collagens [110] (Figure 1). Collagen IV is the most important structural element of BMs, where it binds laminins, nidogens, and other ECM components, and stabilizes the structure [57]. Adipocytes are encircled by a thin BM, and collagen IV is naturally a prominent ECM component in AT [58]. The synthesis of collagen IV in AT appears to vary between developmental stages and in different adipose depots [107,132].

When 3T3-L1 preadipocytes undergo the adipogenic differentiation process, collagen IV production, or, more accurately, the production of α1(IV) and α2(IV) chains, increases significantly alongside some other basal lamina elements [133,144]. Another study reported that the secretion of collagen IV is increased in hypoxia by as much as 10 times, but the mRNA levels of α1(IV) and α2(IV) are not affected by the low oxygen pressure [145]. Interestingly, bone marrow stromal cells do not undergo adipogenic differentiation when cultured on the native structural form of the collagen IV scaffold but do undergo differentiation in a denatured collagen IV matrix. Adipogenic differentiation with denatured collagen IV was shown to occur through integrin αvβ3 integrin signaling [108]. These findings reiterate the important role of matrix remodeling and the balance of ECM-degrading MMP levels and MMP-inhibiting TIMP levels in adipogenesis and suggest that the inhibition of collagen IV denaturation in AT could serve as a strategy for obesity treatment.

In obesity, collagen IV synthesis is upregulated alongside other BM elements in SAT and is associated with the inflammatory and fibrotic factors TGF-β1 and TGF-β3, as well as with insulin resistance [107]. However, in an in vitro setting, TGF-β1 and TGF-β3 stimulated *COL4A1* expression only in endothelial cells isolated from the SAT of patients, and not in isolated adipocytes. After the gastric bypass surgery of severely obese patients and the subsequent weight loss, *COL4A1* expression was downregulated in SAT, and this reduction correlated with improved glucose metabolism parameters, such as insulin resistance assessment (HOMA-IR) in the studied patients [107].

### 3.6. Collagen V

Collagen V is a widely occurring low abundance fibrillar collagen with three different α chains, namely α1(V), α2(V), and α3(V), which form distinct heterotrimeric collagen V molecules. The most common combination which occurs in tissues is composed of two α1(V) chains and one α2(V) chain that assemble into heterotypic collagen fibrils with collagens I and III, thus regulating the fibril geometry [146] (Figure 1). During the differentiation of 3T3-L1 cells and bovine intramuscular preadipocytes, collagen V synthesis rises rapidly at early stages of adipogenesis and, later, the collagen V network undergoes extensive modifications, causing the thickening of fibrils [133,147]. Mice with a *Col5a2* knockout do not survive embryonic development [148], but the *Ubc-CreERT2; Col5a2^fl/fl^* mice with the tamoxifen-induced postnatal ubiquitous ablation of the α2(V) chain present a drastic reduction in dermal and abdominal AT, with small adipocytes and fibrotic abdominal fat pad seams, highlighting the importance of collagen α2(V) for AT maintenance [149].

Mast cells, which gather and possibly mature in AT during obesity and diabetes progression, excrete inflammatory mast cell protease 6 (MCP-6), which has been shown to promote collagen V, specifically *Col5a1*, expression [114]. Collagen V is then said to promote AT fibrosis, and it seems to also suppress preadipocyte differentiation [114]. The co-culturing of adipocytes and M2 macrophages also appears to increase collagen V production in the adipocytes, possibly via TGF-β signaling [38], further suggesting crosstalk between immune cells and adipocytes to regulate collagen V levels in AT. In fibrous AT, collagen V bundles gather around blood vessels in significant quantities [37]. Obese individuals have fewer capillaries but more large vessels in their SAT depots than lean individuals, pointing to impaired angiogenesis in the obese individuals [38]. Collagen V inhibits angiogenesis in endothelial cell cultures, suggesting a link between collagen V levels and impaired angiogenesis in AT [38].

The α3(V) chain has more limited tissue distribution and is found in skeletal muscle, pancreas, and WAT in vertebrates and associates into heterotrimers with one α1(V) and one α2(V) chain [150]. In fact, *Col5a3* is highly expressed in human adipocytes and mouse WAT [115], and its expression is increased in 3T3-L1 cells upon adipogenic differentiation [115]; however, it is decreased when adipogenic differentiation is compromised [134]. Thus, α3(V) seems to have AT-specific functions beyond its structural roles in collagen fibrils [115,146]. Yet, the normal-chow-fed mice with *Col5a3* deletion present only a subtle reduction in SAT and no reduction in abdominal fat. However, feeding them with HFD resulted in a significant decrease in the total body weight of the *Col5a3* knockout females, suggesting a sex-specific resistance to diet-induced obesity in the absence of this collagen chain [115]. *Col5a3* ablation also leads to a significant decrease in insulin-stimulated *Pparg* expression in mouse WAT, further indicating a role of α3(V) in adipocytic differentiation [115].

The same study associated the α1(V), α2(V), and α3(V) chains with the regulation of glucose metabolism [115]. The ablation of α3(V) in mice led to impaired insulin sensitivity and hyperglycemia due to the incorrect deployment of glucose transporter GLUT4 receptors in their WAT and muscles, and this finding was replicated in the 3T3-L1 cell line. However, it should be noted that the knockout *Col5a3* gene led to impaired glucose-stimulated insulin secretion from pancreatic β cells which can also contribute to the observed glucose imbalance in these mutant mice [115].

### 3.7. Collagen VI

Collagen VI is a prominent component of the ECM and usually forms heterotrimers composed of α1(VI), α2(VI), and α3(VI) chains. In addition to these, genes for three other α(VI) chains, i.e., α4(VI), α5(VI), and α6(VI), have been annotated; however, at least the *COL6A4* gene is most likely not functional [121,151] (Figure 1). Collagen α(VI) chains form a separate subfamily among collagens because they assemble into a distinct network of beaded microfilaments that are located in the interphase between BMs and interstitial ECM. It is widely expressed in tissues such as muscle, bone, cartilage, and tissue, as well as in the nervous system, and serves both biomechanical and biochemical functions that regulate cell survival, differentiation, and proliferation [151].

Collagen VI is an abundant component of AT and is produced by adipocytes [121,151,152]. Collagen VI binds to the collagen IV network in the adipocyte BMs, contributing to the rigidity of the pericellular ECM [56]. In humans, collagen VI genes show fat depot-specific expression profiles and are produced in greater quantities in the SAT than in the VAT, and collagen VI mRNA transcripts are enriched in the stromal vascular fraction containing adipocyte precursors over the mature adipocytes [116]. In mice, collagen VI is the most abundant collagen in mature adipocytes [34]. Collagen VI production increases during the adipogenic differentiation of 3T3-L1 cells and bovine intramuscular preadipocytes, and its fiber network undergoes significant thickening during differentiation (Figure 2), which is similar to the collagen V network modification [133,147].

Collagen VI is overexpressed in fibrotic AT and accumulates in areas of pericellular fibrosis around the adipocytes in humans [37]. Increased *COL6A3* expression in obesity restricts fat storage in SAT, which might lead to the lipid accumulation into VAT instead [153]. Moreover, high *COL6A3* expression causes reduced oxygenation of AT, contributing to hypoxia and inflammation in the AT [153]. In obesity, *COL6A3* expression in SAT is higher in humans with insulin resistance than those who are sensitive to insulin [36,119]. It is worth noting that a high BMI (>28) caused high variability in *COL6A3* expression levels [153].

In mice, the *Col6a3* knockout resulted in a reduction in epididymal AT mass but had no effect on the SAT or BAT [117], suggesting significant depot-specific functions. The stromal vascular fraction cells isolated from the SAT of *Col6a3* knockout mice have an impaired adipogenic capacity in vitro [117]. In the in vitro 3T3-L1 model, the deletion of *Col6a3* results in lipid accumulation that is comparable to that of wild type cells, while both *Col6a1* and *Col6a2* knockouts in these cells lead to impaired lipid accumulation. However, impaired lipolysis, which was observed both for *Col6a3* knockout 3T3-L1 cells and *Col6a3* knockout mice, may explain the unaltered lipid accumulation in the 3T3-L1 *Col6a3* knockout cells [117].

The deletion of *Col6a3* in obese *ob/ob* mice leads to improved outcomes and a metabolic phenotype, including lower AT inflammation, improved lipid clearance, and fewer necrotic adipocyte deaths. Adipocyte cell size was found to be larger in *ob*/*ob* and *Col6a3*^−/−^ crosses than in *ob*/*ob* mice expressing *Col6a3*, which probably improved the metabolic phenotype of these obese mice, as adipocytes were able to grow without restrictions [34]. One study reported that, in obese humans, *COL6A3* is upregulated after weight loss and downregulated in obesity, but these fluctuations had no connection to metabolic dysfunction [116]. This is contrary to the results reported by mouse studies and other human studies that support the increased synthesis of collagen α3(VI) chains in obesity [34,119,153].

Animal studies suggest that the α3(VI) chain of collagen VI is the most important α(VI) chain that regulates AT functions. The C-terminal biologically active endotrophin fragment of the α3(VI) chain (Figure 1) has independent roles in AT. During ECM remodeling, endotrophin is cleaved from the parental α3(VI) by BMP-1 protease and does not form a part of the mature collagen VI microfilament network [121]. Endotrophin is associated with a myriad of harmful effects with regard to metabolism, such as increasing the expression of pro-adipogenic genes and causing abnormal increased lipid accumulation and lipolysis [154,155]. It promotes detrimental changes in the AT architecture and leads to a significant increase in serum triglyceride levels during HFD in transgenic mice that overexpress endotrophin specifically in adipocytes [155]. A high endotrophin level has been associated with increased insulin resistance, and, interestingly, blocking the endotrophin function with a specific antibody treatment has been shown to improve metabolic health [155]. Moreover, endotrophin levels were increased in human patients with diabetes [155].

Endotrophin overexpression in adipocytes increases TGF-β signaling and upregulates ECM protein synthesis, including fibrillar collagens, which may account for several of its detrimental effects on metabolism [155]. In another study, endotrophin was also linked to the synthesis of the fibrosis biomarker pro-collagen III, and endotrophin itself has been suggested as a novel biomarker of tissue fibrosis [120,121,156]. Using mice and 3T3-L1 cells, Zhao et al. [154] observed that endotrophin increases the synthesis of various fibrotic proteins in different cell types of AT. For example, in adipocytes, endotrophin induces fibrillar collagen synthesis, whereas, in macrophages, it mainly promotes the expression of the collagen cross-linking enzyme LOX and leads to an increase in AT macrophages and a shift from the M2 phenotype to pro-inflammatory M1 [154,155]. Macrophages appear to be the main mediator through which endotrophin induces AT inflammation, as its pro-inflammatory effects were not observed in adipocytes themselves [154]. In a clinical pilot study where humans with poor glycemic control in T2D were put on a diet and exercise regime, endotrophin levels were high at the start of the study. However, they were significantly lowered due to the change in lifestyle and were correlated with lowered serum glycated HbA1c hemoglobin and urine albumin-creatinine ratio levels, further indicating that α3(VI)/endotrophin expression is associated with metabolic dysfunction [157].

### 3.8. Multiplexin Collagens XV and XVIII

Besides the major AT collagens (types I, III, IV, and VI), other collagen types are also expressed within AT, and some of them play intriguing structural or functional roles. For example, the structurally homologous non-fibrillar BM-associated collagens XV and XVIII of the multiplexin subclass are implicated in adipogenesis and lipid metabolism [158,159] (Figure 1).

Collagen XV is expressed in many cell types of the connective tissue, including adipocytes and fibroblasts [158]. In AT, the roles of type XV collagen are still not very well understood. Nevertheless, in mice, *Col15a1* expression appears to be higher in WAT depots than BAT depots [122]. The adipocyte differentiation process is associated with increased collagen XV synthesis in mice. Collagen XV promotes adipocyte differentiation and inhibits lipolysis, possibly via changes in its DNA methylation and the inhibition of the hormone-responsive cyclic AMP (cAMP)−protein kinase A (PKA) pathway. The cAMP response element binding protein (CREB) suppresses collagen XV expression and, as a result, the collagen XV-dependent promotion of adipocyte differentiation [122]. Moreover, collagen XV levels are significantly increased in obese mice, and this upregulation has been suggested to play a functional role in lipid deposition and adipogenesis [122]. RNA-seq data have demonstrated the involvement of collagen XV in the regulation of abnormal ECM remodeling, which is associated with the induction of adipocyte apoptosis via the collagen XV-activated AMPK pathway [123]. Collagen XV is also involved in the regulation of AT inflammation, as it promotes the polarization of pro-inflammatory M1 macrophages and the upregulation of the endoplasmic reticulum stress-related genes [124].

Type XVIII collagen is a multidomain collagen and heparan sulphate proteoglycan with three alternative forms (short, medium, and long) that differ in terms of domain structure and that have tissue-specific expression patterns and different functional roles [159] (Figure 1). Collagen XVIII production is known to increase during adipogenesis [127,129]. In mice, the effects of collagen XVIII on adipocyte differentiation appear to be largely mediated by the two longest isoforms of collagen XVIII, whose expression is regulated by an alternative internal promoter of the gene, and a specific lack of these isoforms leads to reduced adiposity [126,127]. Mouse embryonic fibroblast isolated from *Col18a1^−/−^* mice or mice specifically lacking the two longest isoforms (the *Col18a1^P2/P2^* mice) have an impaired adipogenic capacity, and the stromal vascular fraction of the epididymal AT of these mice contains more adipocyte progenitor cells than wild-type mice [127]. Wnt/β-catenin signaling is an important adipogenic regulator that suppresses adipogenic progression [143]. Interestingly, the longest isoform of collagen XVIII contains a Frizzled-like domain (Figure 1) that has considerable similarity to the Frizzled receptors of the Wnt ligands [159], suggesting that collagen XVIII may regulate adipogenesis through the Wnt/β-catenin pathway. In fact, immunoprecipitation analysis indicates the binding of the Frizzled-containing domain of collagen XVIII with the Wnt10b [127]. An impaired capacity of *Col18a1*^−/−^ and *Col18a1^P2/P2^* mutants to store lipids in the AT causes ectopic lipid accumulation and dyslipidemia, which leads to increased serum triglyceride levels and higher fat accumulation in the liver compared to wild-type mice [126,127].

Hypertriglyceridemia has also been observed in humans with mutations in the *COL18A1* gene [125]. Multiple single-nucleotide polymorphisms (SNP) in *COL18A1* are associated with obesity in T2D and with circulating lipid contents [128,129,130]. Moreover, collagen XVIII synthesis in VAT is associated with circulating free fatty acids in obesity, and a genetic linkage analysis shows an association between chromosome 21, where *COL18A1* is located, and the familial combined hyperlipidemia-triglyceride trait, as well as increased serum triglycerides in hypertensive pedigrees [127,160]. Knobloch syndrome patients carrying a null mutation of *COL18A* have increased serum triglyceride levels after fasting and show reduced activity and mass of plasma lipoprotein lipase (LPL), which cleaves fatty acids from circulating lipoproteins [125,161]. The heparan sulphate side chains of collagen XVIII are suggested to carry LPL from the ECM to its receptor on the endothelial cell membrane, and a lack of this collagen may lead to the retention of LPL in the subendothelial matrix, leading to dysregulation in blood lipid profiles and dyslipidemias [125,161].

Furthermore, a type XVIII collagen knockout in mice causes metabolic complications, specifically reduced insulin sensitivity and glucose tolerance, which are most likely caused by the aforementioned lack of adiposity [126]. Interestingly, collagen XVIII deficiency also leads to increased heat production, probably due to the increased thermogenesis in mouse BAT, as well as changes in BAT composition [126]. BAT serves an intriguing function in AT-related lipid regulation. It appears that the increased activation of BAT thermogenesis will positively impact the lipid profile, lowering the risk of atherosclerosis [162,163]. Interestingly, *Col18a1*^−/−^ mice displayed an improved triglyceride profile in circulation at cold temperatures, likely due to the activated lipid uptake and non-shivering thermogenesis in their BAT [126].

### 3.9. Other Collagens

Studies on AT-associated collagens have been focused on fibrillar and BM collagens and collagen VI, but a few transcriptome analyses have also revealed interesting expression patterns in AT for other collagen types. For example, the fibril-associated collagen XII and non-fibrillar short-chain collagen VIII were found to be upregulated in the adipocytes of obese individuals. Here, a high expression of *COL12A1* is strongly associated with the amount of LDL, the “bad cholesterol,” whereas low *COL12A1* is related to improved insulin sensitivity [47]. Collagen VIII is an understudied collagen, but it has been suggested to have functions related to endothelial cells, smooth muscle cells, and myofibroblasts [164], and, thus, probably also in the AT vascularization and fibrosis. Another microarray study revealed the downregulation of *COL14A1* when human mesenchymal stem cells were induced to differentiate to mature adipocytes in vitro [165]. This deviates from the findings of studies on 3T3-L1 cells that have reported that collagen XIV, and, specifically, its FN type III domain, triggered adipogenic differentiation in these cells [166]. Finally, collagen XXIV was upregulated in the VAT and skeletal muscles of HFD-fed mice, as well as in the VAT, but not the SAT, of obese diabetic human subjects compared to lean controls, suggesting a pathogenic function of this collagen in T2D and obesity [131].

## 4. Conclusions and Future Perspectives

**Figure 3:**
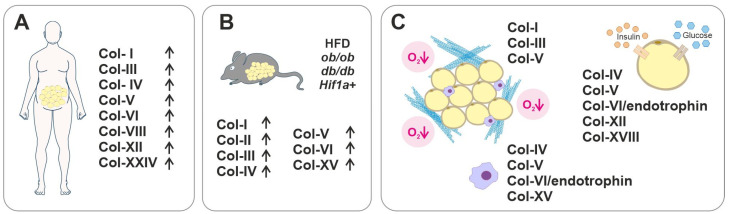
Summary of collagens in AT dysfunction. (**A**) Upregulated collagens (arrows) in the WAT of obese humans. (**B**) Upregulated collagens (arrows) in the WAT of mice fed with a high-fat diet (HFD), or in obese *ob/ob* or diabetic *db/db* mouse models, or in the *Hif1a+* mice with constitutively active HIF-1α. (**C**) Left: Key collagens forming large collagen bundles (blue) in hypoxic (pink) and fibrotic AT. Collagens which are reported to be associated with insulin resistance (right upper corner), or with AT inflammation (bottom, a macrophage depicted).

AT is a highly active tissue that plays a vital role in metabolism and the general health of the entire body. AT-resident ECM and its major collagen components help guide AT growth by providing a supportive and modifiable scaffold and acting as a restrictive barrier. AT ECM is capable of undergoing a myriad of changes in response to environmental and nutritional cues. Beyond their structural role, AT collagens play significantly functional roles in adipocyte maturation and whole-body metabolism (Figure 2 and Figure 3). The relatively recently discovered ECM component, collagen α3(VI)-derived endotrophin fragment, for example, has emerged as an important regulator of adipogenesis and AT function. High endotrophic levels are significantly associated with many chronic diseases including T2D, vascular, and kidney diseases, and it was proposed to serve as a new prognostic biomarker for such metabolic disorders [153,156]. In addition to the widely studied fibrillar collagens, the BM-associated collagen XVIII appears to majorly contribute to regulating AT development and functions and the related metabolic complications such as dyslipidemia (Figure 3).

Collagen and ECM modulation can be a powerful approach to affect whole AT homeostasis and, thus, research on AT collagens can open interesting avenues into the development of novel anti-T2D and obesity therapies. However, their complex dual role in both the physiology and pathology of AT is a challenge. Further research into the diversity of AT collagens is expected to reveal novel structural and functional roles for collagens. Understanding the developmental and physiological roles of AT collagens versus the pathological effects of fibrotic ECM in AT, and its effects on the insulin sensitivity of adipocytes and other AT cells are central questions to be answered in studies focusing on AT ECM. Furthermore, how much do posttranslational modifications of collagens affect their relevant functions, and do LOX- and transglutaminase-mediated crosslinking alter their impact from positive to negative? How do other ECM components such as fibronectin affect collagenous AT ECM development and functioning? Also, what are the cellular mechanisms that promote the expression and assembly of collagens that are necessary for AT health versus those cues that initiate fibrosis? What is the role of ECM in general, and collagens in particular, in modulating the heterogeneity and plasticity of adipocytes? These and many other questions must be carefully addressed in future AT ECM studies.

## Figures and Tables

**Table 1:** Expression of selected collagens in dysregulated human and mouse adipose tissue (AT).

Collagen	Pathological Condition	Expression/Manifestation	References
I	Fibrotic AT T1D	Increased expression in obese AT compared to lean subjects Decreased level of crosslinked telopeptide in the serum of T1D patients with retinopathy	[34,37,111,112]
II	T2D	Increased expression in epididymal AT in diabetic (*db/db*) mice	[34]
III	Fibrotic AT T1D T2D	Increased expression in obese AT compared to lean subjects and in patients with T1D with retinopathy Increased levels of procollagen aminopeptide in patients with T2D and progressing diabetic nephropathy	[37,111,113]
IV	T2D	Increased *Col4a1* and *Col4a2* expression in the WAT of diabetic mice Downregulation of *COL4A1* in SAT after gastric bypass and improvement of HOMA-IR	[34,107]
V	Fibrotic AT Impaired glucose metabolism Insulin resistance	Increased expression in the WAT of diabetic mice Increased expression in obesity; accumulation in fibrotic areas, especially around large blood vessels. Fibrotic promotion causes insulin resistance Lack of *Col5a3* leads to impaired glucose metabolism	[34,38,114,115]
VI	Fibrotic AT Altered glucose metabolism	Increased expression in obese patients associates with pericellular fibrosis Biomarker in AT fibrosis Conflicting results in glucose metabolism; insulin resistance vs. improved glucose metabolism Increased expression in obese/diabetic mice while downregulated in obese humans	[34,37,116,117,118,119,120,121]
VIII	Obesity	In the twins study, an increased expression of *COL8A2* was found in the heavier twin	[47]
XII	Insulin resistance	In the twins study, *COL12A1* expression positively associated with LDL cholesterol, and low expression associated with increased insulin sensitivity	[47]
XV	Obesity	Increased expression in AT in HFD-induced obesity in mice Regulates adipocyte apoptosis and inflammation in AT	[122,123,124]
XVIII	Visceral obesity in T2D Dyslipidemia Lipodystrophy	Specific SNPs associate with obesity in patients with T2D (c.1136C > T) and with abnormal circulating lipid content (c.331G > A, p.Gly111Arg) Patients with Knobloch syndrome due to *COL18A1* null mutation have fasting hypertriglyceridemia Lack of *Col18a1* in mice causes lipodystrophy, T2D, and increased serum triglyceride levels Expression of long isoforms of collagen XVIII in visceral fat positively correlates with free fatty acid levels in the plasma	[125,126,127,128,129,130]
XXIV	T2D	Increased expression in insulin-resistant obese VAT	[131]

Abbreviations: AT—adipose tissue; HFD—high-fat diet; HOMA-IR—homeostatic model assessment for insulin resistance; LDL—low-density lipoprotein; SAT—subcutaneous adipose tissue; T1D/T2D—type 1/2 diabetes; WAT—white AT.

## Data Availability

No new data were created or analyzed in this article. Data sharing is not applicable to this article.
